# Risk factors for postoperative myasthenia gravis in patients with thymoma without myasthenia gravis: A systematic review and meta-analysis

**DOI:** 10.3389/fonc.2023.1061264

**Published:** 2023-02-08

**Authors:** Mingbo Tang, Yifeng Shao, Junxue Dong, Xinliang Gao, Shixiong Wei, Jianzun Ma, Yang Hong, Zhiqin Li, Taiyu Bi, Yipeng Yin, Wenyu Zhang, Wei Liu

**Affiliations:** ^1^ Department of Thoracic Surgery, The First Hospital of Jilin University, Changchun, Jilin, China; ^2^ Laboratory of Infection Oncology, Institute of Clinical Molecular Biology, Universitätsklinikum Schleswig-Holstein (UKSH), Christian Albrechts University of Kiel, Kiel, Germany

**Keywords:** postoperative myasthenia gravis, thymoma, operation, thymectomy, AChR-ab

## Abstract

**Introduction:**

According to the principle, thymomas combined with myasthenia gravis (MG) require surgical treatment. However, patients with non-MG thymoma rarely develop MG and early- or late-onset MG after surgery is called postoperative MG (PMG). Our study used a meta-analysis to examine the incidence of PMG and risk factors.

**Methods:**

Relevant studies were searched for in the PubMed, EMBASE, Web of Science, CNKI,and Wanfang databases. Investigations that directly or indirectly analyzed the risk factors for PMG development in patients with non-MG thymoma were included in this study. Furthermore, risk ratios (RR) with 95% confidence intervals (CI) were pooled using meta-analysis, and fixed-effects or random-effects models were used depending on the heterogeneity of the included studies.

**Results:**

Thirteen cohorts containing 2,448 patients that met the inclusion criteria were included. Metaanalysis revealed that the incidence of PMG in preoperative patients with non-MG thymoma was 8%. Preoperative seropositive acetylcholine receptor antibody (AChR-Ab) (RR = 5.53, 95% CI 2.36 – 12.96, P<0.001), open thymectomy (RR =1.84, 95% CI 1.39 – 2.43, P<0.001), non-R0 resection (RR = 1.87, 95% CI 1.36 – 2.54, P<0.001), world health organization (WHO) type B (RR =1.80, 95% CI 1.07 – 3.04, P= 0.028), and postoperative inflammation (RR = 1.63, 95% CI 1.26 – 2.12, P<0.001) were the risk factors for PMG in patients with thymoma. Masaoka stage (P = 0.151) and sex (P = 0.777) were not significantly associated with PMG.

**Discussion:**

Patients with thymoma but without MG had a high probability of developing PMG. Although the incidence of PMG was very low, thymectomy could not completely prevent the occurrence of MG. Preoperative seropositive AChR-Ab level, open thymectomy, non-R0 resection, WHO type B, and postoperative inflammation were risk factors for PMG.

**Systematic Review Registration:**

https://www.crd.york.ac.uk/PROSPERO/, identifier CRD42022360002.

## 1 Introduction

Myasthenia gravis (MG) is an autoimmune disease caused by impaired acetylcholine receptor (AChR) transmission at the neuromuscular junction, and it is characterized by partial or systemic skeletal muscle weakness and fatigue, which are closely related to the thymus. According to reports, approximately 8.5% – 15% of patients with MG are complicated with thymoma, while approximately 15% – 40% of patients with thymoma are complicated with MG (1–5).

Thymoma is a malignant tumor originating from thymic epithelial cells. Although thymoma has no clinical symptoms on its own, it is frequently associated with various immune diseases, including MG, and most researchers recommend surgical resection once thymoma is diagnosed (6). The possibility of MG occurrence should be investigated in the preoperative evaluation of patients with thymoma, as perioperative management of associated MG is critical (7). Notably, in clinical practice, we rarely encounter early- or late-onset occurrences of MG in patients with non-MG thymoma after surgery, clinically called “postoperative MG (PMG).” The pathogenesis of PMG may differ from that of primary MG, which remains unclear.

In previous studies of non-MG thymomas, the incidence rate of PMG ranged widely from 0.9% to 28%, and the conclusions on the risk factors of PMG in patients with non-MG thymoma are controversial between studies (2, 6, 8, 9). Generally, PMG has received little attention due to its low incidence, and the limited number of studies available are mostly single-center retrospective studies. To our knowledge, no systematic review or meta-analysis has yet provided evidence-based medical findings. However, it is important to clarify the clinicopathological characteristics and risk factors of patients with PMG, not only for perioperative management but also for investigating the relationship between thymoma and MG and the development process of MG. Therefore, this study aimed to explore the incidence and risk factors of PMG in patients with non-MG thymoma through a meta-analysis.

## 2 Methods

This study adhered to the PRISMA guidelines and developed exclusion criteria based on the PICOS model. The PROSPERO registration number is CRD42022360002.

### 2.1 Literature search

PubMed, EMBASE, Web of Science, Wanfang, and CNKI databases were searched. The Medical subject headings (MeSH) terms, Emtree terms, and keywords used are as follows: myasthenia gravis, thymoma, thymus tumor, thymectomy, thymomectomy, and thymothymectomy. All publications, regardless of the article type, were carefully screened to determine eligibility. Furthermore, we filtered references to relevant publications that were found in our search for eligible studies.

### 2.2 Study selection

Two physicians in our department independently read and reviewed the literature and finally reached a consensus on inclusion in the study. When consensus could not be reached, a third researcher participated in the study inclusion process. We selected published articles that met the following criteria: (1) patients with a pathological diagnosis of thymoma, (2) patients who underwent thymectomy or extended thymectomy, (3) patients who developed MG after surgery, and (4) studies written in English or Chinese. The exclusion criteria were as follows: (1) symptoms of MG before surgery, (2) repeated paper retrieval, (3) studies that did not include the research indicators required by meta-analysis, (4) lack of research data that could not allow data extraction for meta-analysis, and (5) case reports, reviews, and the full text of literature that could not be obtained.

### 2.3 Data extraction and literature quality evaluation

The two authors independently extracted data from eligible studies and aggregated them using a data extraction table, which was not mutually communicative. Studies that did not meet the inclusion criteria were excluded following the review of the title and abstract. Subsequently, the researchers read the studies’ full text and screened them further. The quality of the literature was assessed using the Newcastle Otava Scale (NOS) (10), which consisted of three blocks with a total of eight items (for cohort and case-control studies). Additionally, the quality of the literature was evaluated using the semi-quantitative principle of the star system in three parts (study population selection, comparability, exposure assessment, and outcome assessment).

### 2.4 Data analysis

Data were analyzed using Stata statistical software version 15 (StataCorp., College Station, TX, USA). Heterogeneity between studies was determined using the Q test, and when the I^2^ metric was *P* ≥ 0.10 in the Q test or I^2^ < 50%, the fixed-effect model (the Mantel Haenszel method) was applied (11). Otherwise, a random effects model (DerSimonian and Laird) analysis was conducted (12). In the test, I^2^ < 50% was considered as having no heterogeneity, and the fixed-effects model was used for pooled analysis. I^2^ > 50% was regarded as having heterogeneity, and the random effects model was used for pooled analysis. Additionally, we assessed the probability of publication bias using Peters regression tests (13). Moreover, the Egger test and funnel plot were utilized to evaluate publication bias, and *P* < 0.05 was considered to indicate publication bias. Statistical significance was defined as a two-tailed *P* < 0.05.

## 3 Results

### 3.1 Study characteristics of the selected studies

**Figure 1:**
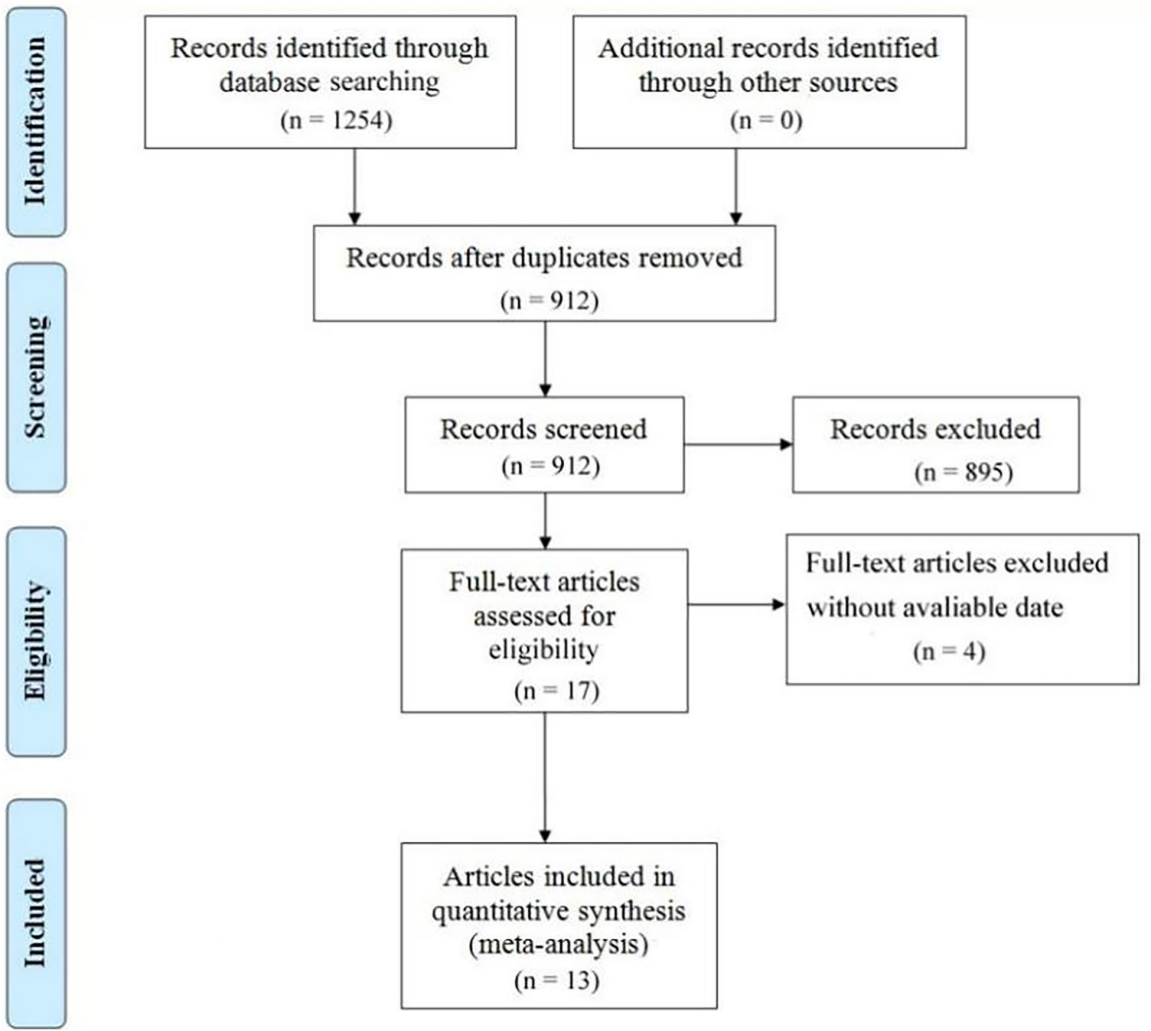
Study flow diagram.

The initial study included 1,254 potential articles, of which 912 were available after eliminating duplicate data from each database. Furthermore, after reading the titles and abstracts, 895 studies were excluded because they were irrelevant to our study design. The other 17 full text studies were carefully evaluated for eligibility, and four were excluded because no data were available. Ultimately, 13 studies were included in the analysis, and all 13 met the allocation concealment criteria (**Figure 1**). **Table 1** shows the summary of this study’s characteristics of the selected articles, including the author’s name, publication year, patient source (country), duration, sample size, number of PMG, age of patients with MG, sex of patients with PMG, the interval between surgery and PMG, risk factors, and NOS of studies.

**Table 1:** Characteristics on risk factors for PTMG.

Study, year	Country	Duration	Study-design	Sample size	No. of MG	MG patients' age (mean):	MG patients' Gender (M:F)	Interval	Risk factors	NOS
Li 2004	China	1963-1998	Retrospective	127	15	median:42	6:09	mean:33.9 months	Gender, Masaoka-stage	6
Kondo 2005	Japan	1990-1994	Retrospective	827	8	50.5±15.0	1:07	6-days-45 months	NR	7
Nakajima 2008	Japan	1994-2007	Retrospective	55	5	45.4±10.53	3:02	3-46- months	Preoperative ARAB, WHO histology, Masaoka stage	8
Yamada 2015	Japan	1991-2011	Retrospective	123	10	57.1±15.89	7:03	3-2859 days	Preoperative ARBA, Gender, WHO histology, Masaoka stage, Surgical-approach, Tumour complete resection	8
Sun 2010	China	1990-2008	Retrospective	125	6	43±12.13	3:03	1 week- 31 months	Preoperative ARAB	7
Qian 2017	China	2002-2015	Retrospective	126	9	48.67±11.110	4:05	10-1860 days	Gender. WHO histology, Masaoka stage, Surgical approach, Pneumonia, Tumour complete resection	8
Zhao 2017	China	2008-2016	Retrospective	379	35	53.24±5.67	13:22	NR	Gender, Surgical approach, Pneumonia, Tumour complete resection	7
Mineo 2018	Italy	1987-2013	Retrospective	104	8	42.88±13.32	3:05	median:33 months	Preoperative ARAB, Gender, WHO histology, Masaoka stage	7
Xu 2020	China	2019-2020	Retrospective	92	110	51.1±10.00	5:06	NR	Gender, WHO-histology, Surgical-approach, Pneumonia	7
Zhang 2020	China	2004-2016	Retrospective!!	229	19	median:33	9:10	2-730 days	Gender, WHO histology, Masaoka stage, Surgical- approach, Pneumonia, Tumour complete resection	7
Kim 2021	Korea	2008-2017	Retrospectivel	191	8	56.5±10.17	2:06p	18 days-108 months	Preoperative ARAB, Gender, WHO histology, Masaoka stage, Surgical approach, Tumour complete resection	8
Marcuse 2021	Netherlands	2004-2018	Retrospectivel:	44	10	54.4 ±9.64	3:07	1 day-72- months	Gender, WHO thistologl	8
Nabe 2021	Japan	2013-2020	Retrospective!	26	3	NR	NR	NR	Preoperative ARAB	7

ARAB, anti-acetylcholine- receptor binding antibody; MG, myasthenia gravis; Interval, interval between thymectomy and onset of postoperative MG; NR, not reported.

### 3.2 Meta-analysis of PMG incidence in preoperative patients with non-MG thymoma

**Figure 2:**
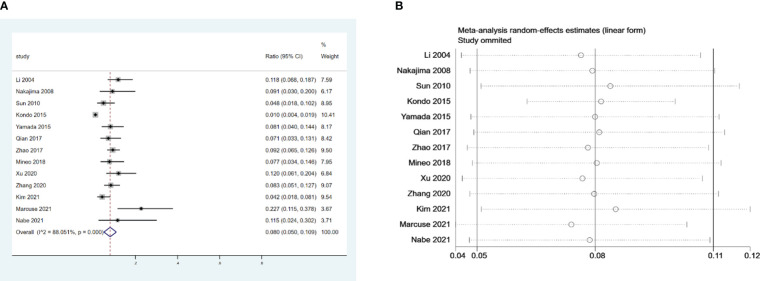
**(A)** Meta-analysis of the incidence of PMG in patients with thymoma without preoperative MG. **(B)** Sensitivity-analysis of the incidence of PMG in patients with thymoma without preoperative MG.

We examined 13 retrospective studies that included 2,448 patients and investigated the incidence of PMG (1, 6, 14–24). Our meta-analysis revealed that the incidence of PMG in preoperative patients with non-MG thymoma was 8% (95% confidence intervals (CI), 5% – 10.9%; I2 = 88.1%, **Figure 2A**). We conducted a meta-analysis of the pooled results, and our conclusions are robust as shown in **Figure 2B**.

### 3.3 Meta-analysis of the influence of preoperative acetylcholine receptor antibody level on PMG

**Figure 3:**
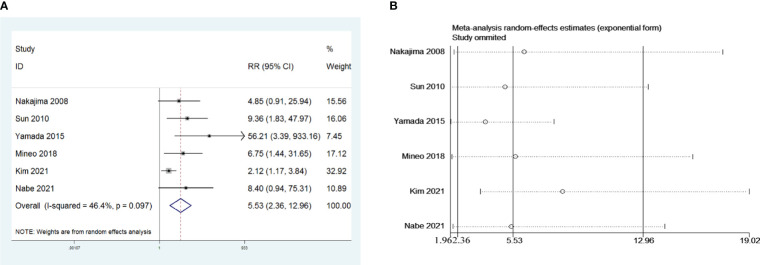
**(A)** Meta-analysis of the influence of preoperative AChR-Ab level on PMG. **(B)** Sensitivity-analysis of the influence of preoperative AChR-Ab level on PMG.

Six retrospective studies that included 624 patients were selected to investigate the influence of preoperative AChR-Ab levels on PMG. The meta-analysis indicated that seropositive AChR-Ab was a risk factor for PMG in patients with thymoma without preoperative MG (risk ratio (RR) = 5.53, 95% CI 2.36 – 12.96, and P<0.001; I2 = 46.4%, **Figure 3A**). We conducted a meta-analysis of the pooled results, and our conclusions are robust as shown in **Figure 3B**.

### 3.4 Meta-analysis of the influence of operative approach on PMG

**Figure 4:**
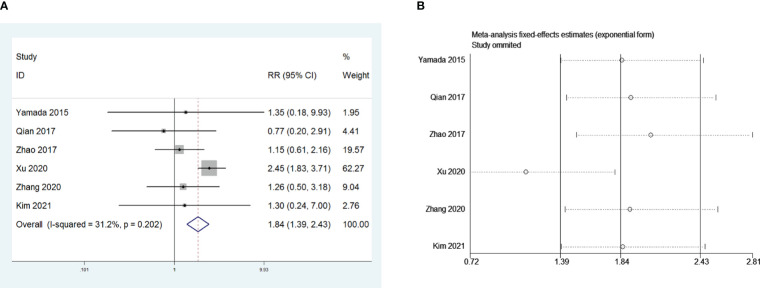
**(A)** Meta-analysis of the influence of operative approach on PMG. **(B)** Sensitivity-analysis of the influence of operative approach on PMG.

Five retrospective studies that included 1,140 patients were included to investigate the influence of open thymectomy versus video-assisted thoracoscopic surgery (VATS) thymectomy on PMG. The meta-analysis indicated that open thymectomy was a risk factor for PMG development in patients with thymoma without MG preoperative (RR = 1.84, 95% CI 1.39 – 2.43, and P<0.001; I2 = 31.2%, **Figure 4A**). We conducted a meta-analysis of the pooled results, and our conclusions are not robust as shown in **Figure 4B**. We found a larger effect on the combined effect size in the study by Xu et al. (14).

### 3.5 Meta-analysis of the influence of complete (R0) thymoma resection on PMG

**Figure 5:**
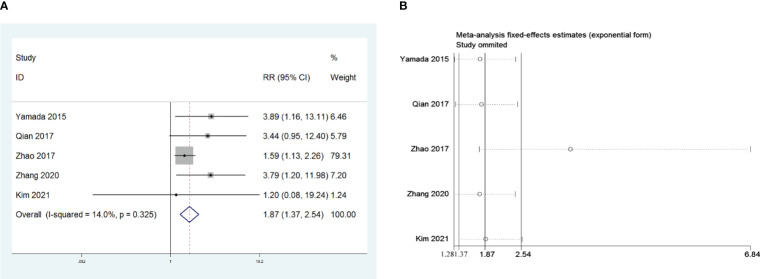
**(A)** Meta-analysis of the influence of complete thymoma resection on PMG. **(B)** Sensitivity-analysis of the influence of complete thymoma resection on PMG.

Five retrospective studies that included 1,048 patients were recruited to investigate the influence of R0 resection on PMG. The meta-analysis indicated that compared with R0 resection, non-R0 resection was a risk factor for PMG in patients with thymoma without MG (RR = 1.87, 95% CI 1.36 –2.54, and *P*<0.001; I^2^ = 14.0%, **Figure 5A**). We conducted a meta-analysis of the pooled results, and our conclusions are not robust as shown in **Figure 5B**. We found a larger effect on the combined effect size in the study by Zhao et al. (16).

### 3.6 Meta-analysis of the influence of World Health Organization classification on PMG

**Figure 6:**
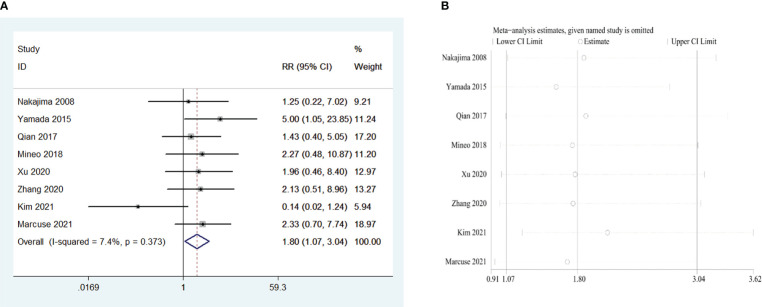
**(A)** Meta-analysis of the influence of World Health Organization classification on PMG. **(B)** Sensitivity-analysis of the influence of World Health Organization classification on PMG.

Eight retrospective studies that included 964 patients were examined to investigate the influence of the WHO thymoma classification on PMG. The meta-analysis indicated that, compared with WHO type A and AB, type B (including B1, B2, and B3) is a risk factor for PMG in patients with thymoma without MG (RR = 1.80, 95% CI 1.07 – 3.04, and P = 0.028; I2 = 7.4%, **Figure 6A**). We conducted a meta-analysis of the pooled results, and our conclusions are robust as shown in **Figure 6B**.

### 3.7 Meta-analysis of the influence of postoperative pneumonia on PMG

**Figure 7:**
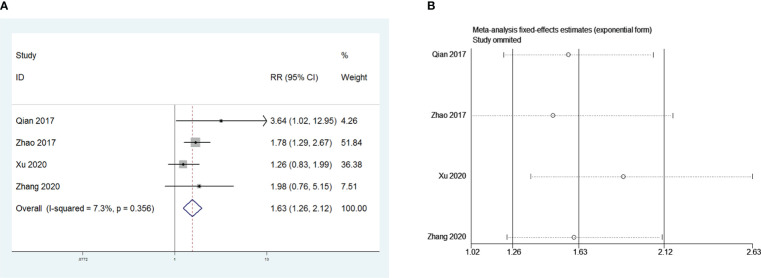
**(A)** Meta-analysis of the influence of postoperative pneumonia on PMG. **(B)** Sensitivity-analysis of the influence of postoperative pneumonia on PMG.

Four retrospective studies that included 826 patients were included to investigate the influence of postoperative pneumonia on PMG. The meta-analysis showed that postoperative pneumonia was a risk factor for PMG in patients with thymoma without MG (RR = 1.63, 95% CI 1.26 – 2.12, and P<0.001; I2 = 7.3%, **Figure 7A**). We conducted a meta-analysis of the pooled results, and our conclusions are robust as shown in **Figure 7B**.

### 3.8 Meta-analysis of the influence of sex and Masaoka stage on PMG

**Figure 8:**
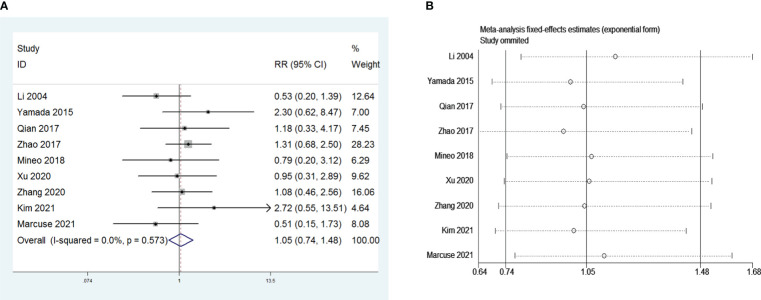
**(A)** Meta-analysis of patients’ gender on PMG. **(B)** Sensitivity-analysis of patients’ gender on PMG.

**Figure 9:**
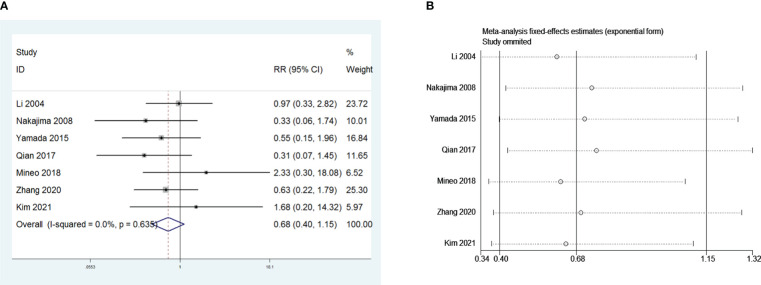
**(A)** Meta-analysis of Masaoka stage on PMG. **(B)** Sensitivity-analysis of Masaoka stage on PMG.

Nine and seven retrospective studies that included 1,415 and 955 patients, respectively, were examined to investigate the influence of sex and the Masaoka stage on PMG. The meta-analysis showed no significant relationship between PMG and sex or Masaoka stage of patients (*P* = 0.151 and 0.777, respectively, **Figures 8A** and **9A**). We conducted a meta-analysis of the two pooled results, and our conclusions are robust as shown in **Figures 8B** and **9B**.

## 4 Discussion

MG is an autoimmune disease caused by a reduction in the number and density of AChRs in the muscle connection, which can be diagnosed based on medical history, physical examination, myogram, and autoantibodies in the serum (25). The early clinical manifestations of MG primarily include ptosis, diplopia, facial, neck, and proximal muscle weakness, and the symptoms are frequently relieved at sunrise and aggravated at sunset, while the main manifestations of MG crisis are respiratory distress and dysphagia. Previous studies have shown that 70% of patients with MG have thymic hyperplasia and 10 – 12% have a thymoma; therefore, the disease is closely related to the thymus.

From a pathophysiological perspective, the thymus is a unique primary lymphoid organ that mediates T cell maturation. Thymomas are composed of numerous CD4+CD8+ double-positive T cells (26), and the decreased HLA-DR molecular level expressed by thymoma tumor epithelial cells may affect the positive selection of CD4+CD8-T cells. Thymomas emerge from the cortical suprortex and lack a functional medulla, where mature antigen-presenting cells participate in negative selection (27). Additionally, patients with thymomas are deficient in the expression of autoimmune regulatory factor (AIRE), a transcription factor in the medulla of the thymus that drives ectopic expression of peripheral tissue-specific autoantigens, including the AChRα subunit. In 95% of patients with thymoma, the deficiency in AIRE expression may be one of the reasons for the autoimmune-related pathways disorder (28, 29). Furthermore, surgical resection is recommended regardless of MG since thymoma is a low-grade malignant tumor with potential invasion and metastatic abilities (30).

Previous studies on patients with thymomas have primarily focused on the recurrence of thymoma and the impact of various surgical procedures on the prognosis of patients with thymoma and on the risk factors of postoperative MG crisis in patients with thymoma. Moreover, the incidence of PMG in patients with thymoma without MG is very low, and the related published studies are limited, with the incidence of PMG ranging from 0.9% to 28% (2, 6, 8, 9). In a single-group meta-analysis, the incidence of PMG after non-MG thymoma surgery was 8%.

Our study revealed that preoperative positive serum AChR-Ab levels were a significant risk factor for PMG development. Continuous antigen-driven B-cell proliferation and selection in the thymic germinal centers of patients with MG provided a rich source of AChR-specific plasma cell precursors (31). Additionally, AChR-Ab was of great reference significance in the diagnosis of MG, and the specificity of AChR-Ab seropositivity for MG was approximately 100% (32). Furthermore, when thymoma diagnosis was suspected, the patients were advised to routinely undergo AChR-Ab testing even without MG symptoms (33, 34). Regarding the specific concentration titer, Watanabe et al. reported that the level of AChR-Ab greater than 100 nmol/L was an independent risk factor for PMG in patients with MG (35). However, we observed that different detection methods might lead to variations in baseline titer levels. Therefore, our study suggested that clinicians should carefully monitor patients when the preoperative AChR-Ab is seropositive, as it not only indicates an increased incidence of PMG but also influences other medical decisions of clinicians. For instance, patients with AChR-Ab-positive should take active preventive measures, such as using the appropriate medication, fewer muscle relaxants in anesthetics, and controlled ventilation during thymectomy (34).

Moreover, studies have suggested that chemotherapy and immune checkpoint inhibitors may need to be reconsidered in patients with AChR-Ab-positive thymoma (36–38). Additionally, thymectomy can improve the clinical outcome of patients with non-thymoma AChR-Ab positive MG; therefore, thymectomy is recommended for all patients with MG and AChR-Ab positivity (39, 40). Summarily, we suggested that when patients are positive for preoperative AChR-Ab, strict preoperative evaluations should be performed jointly with neurologists to ensure perioperative safety and prevent the development of early- and late-onset PMG.

Surprisingly, our study indicated that PMG was more likely to occur after open thymectomy than after VATS thymectomy, and the safety and feasibility of VATS for patients with thymoma have been effectively verified (13, 28). However, in our subsequent sensitivity analysis, we found that the study by Xu et al. had a greater impact on the robustness of this conclusion of ours. When we reviewed the study by Xu et al, we found that their study had the smallest sample size of all the studies included in the operative approach, which may introduce a larger bias. Thus, we should treat this conclusion with caution. Whether the operative approach influences the occurrence of PMG or not needs to be verified by prospective studies that include larger sample sizes. A meta-analysis showed that, compared with thoracotomy, VATS has advantages in terms of blood loss, transfusion rate, average chest tube intubation time, duration of hospital stay, and complication rate, while no significant difference in 5-year overall survival, disease-free survival, and recurrence-free survival rates was observed (29). If subsequent studies prove that minimally invasive surgery does reduce the incidence of PMG, this would be an additional advantage of VATS. Although the mechanism was unclear, we suspected the following might be responsible for this result. First, open thoracotomy surgery caused more trauma to the body and more severe muscle damage, which increased the risk of postoperative MG crisis and led to an increased incidence of early onset PMG. Second, patients who were chosen to undergo open thoracotomy might have had a higher degree of tumor invasion and malignancy and worse WHO classification, leading to an increased probability of late-onset PMG. However, we could not determine whether the timing of PMG development after open thymectomy was more likely to be at the early or late-onset because of the lack of raw data. In addition, greater trauma to the open thoracotomy leads to an increased risk of postoperative infection, and our study showed that postoperative lung infection is another risk factor for PMG. Notably, the levels of inflammatory factors in patients with postoperative pneumonia increased, and the autoimmune response was activated, leading to the occurrence of pathogenic AChR-Ab and the induction of PMG. Simultaneously, it was observed that postoperative pneumonia could also affect respiratory function, leading to pulmonary ventilation dysfunction and difficulty in expectoration, as frequent expectoration leads to respiratory muscle fatigue, thereby increasing the risk of MG.

Furthermore, our analysis revealed that thymoma without complete (R0) resection was a risk factor for PMG development, which was not surprising. Although in the subsequent sensitivity analysis, we found that the study by Zhao et al. had a greater impact on the conclusions, it did not affect the trend of the final conclusions in terms of the graphs. Some researchers believe that the source of AChR-Ab in MG after thymectomy is mainly distributed in the germinal center of residual thymus tissue in the mediastinal region (41, 42). Previous studies confirmed that thymomas release mature autoantigen-specific T cells to the periphery, and the abnormal structure and function of the remaining thymus tissue after incomplete resection can cause the loss of autoreactive T cell clones’ inhibition, leading to autoantigen tolerance disorder and thus the PMG development (43, 44). Additionally, Michalska et al. believed that residual tumor tissue leads to the persistence of postoperative autoimmune response and that PMG may be caused by residual neuromuscular connection damage combined with surgical stress response (45). Concurrently, we considered that many patients with incomplete tumor resection had thymoma invading vital organs, such as the aorta, pulmonary artery, trachea, and phrenic nerves, and could only perform palliative resection. Such thymomas frequently exhibit more aggression and have worse WHO classifications, which could also lead to an increased possibility of PMG development. A prospective autopsy study observed ectopic thymic tissue in 64% of 50 cadavers even after extended thymectomy (46). Notably, the relevant investigations included in this study involved various surgical procedures for thymoma, as shown in **Supplementary Table 1**. However, our study could not analyze the specific surgical method, such as thymectomy and thymomectomy, as a factor affecting PMG, and this was because the enrolled studies differed in their classification of the surgical procedures. In addition, we could not extract the original data for analysis because most of the included studies did not analyze the surgical procedure as a factor affecting PMG. Some researchers believe that thymectomy should be performed, while other authors have concluded that thymectomy may not always be required for patients with early stage thymoma without MG (47, 48). Nevertheless, R0 resection for thymoma is undoubtedly necessary in any case (2, 6), and studies have shown that late-onset PMG is often associated with (23) thymoma recurrence (49–51). Forquer et al. suggested that patients with thymomas should undergo postoperative chemoradiotherapy, especially those with invasive thymomas or non-R0 resections (52). Therefore, if surgical treatment cannot completely eliminate the tumor, preoperative radiotherapy and chemotherapy should be performed before surgical indications are considered, not only for oncological reasons but also to prevent the occurrence of PMG.

WHO types B1, B2, and B3 thymoma histology were observed to be another risk factor for the development of MG, possibly because of the different biological characteristics among the varying histological types. Thymomas of types A and AB were considered relatively benign tumors compared with type B thymomas (53). A study using a global database demonstrated that the tissue type of thymoma had a significant impact on recurrence, and the 5-year recurrence rate was 4%, 2%, 8%, 13%, and 14% for types A, AB, B1, B2, and B3, respectively (54), and recurrence of thymoma is also one of the hypotheses for PMG as mentioned above. In addition, previous studies have shown that thymoma size is related to the occurrence of MG after thymectomy (22). However, the current staging criteria for thymomas did not consider tumor size (55).

Notably, this study analyzed PMG broadly and did not distinguish whether PMG occurred immediately after surgery, delayed months, or even years after surgery, and this was because the time of PMG occurrence was not indicated in the original studies included in our investigation which the period of PMG occurrence in these studies was extensive (1–108 months). We believe that MG occurring immediately after surgery and late-onset PMG may have completely different mechanisms. Perioperative MG may be more closely related to surgical stimulation and stress, whereas late-onset MG may be more closely related to tumor recurrence and changes in the immune environment (49). Nevertheless, the patients with thymoma included in this study did not exhibit any symptoms of MG before surgery but developed PMG after surgery. Through meta-analysis, risk factors for PMG can be established in the perioperative period, which can provide better guidance for perioperative management and postoperative adjuvant therapy.

## Data availability statement

The original contributions presented in the study are included in the article/**Supplementary Material**. Further inquiries can be directed to the corresponding author.

## Author contributions

WL designed the study. MT and YS collected the data. MT did the data analysis, and YS wrote the first version of the manuscript. All the other authors revised the original draft. All authors contributed to the article and approved the submitted version.
