# Supplementary material for: Risk factors for postoperative myasthenia gravis in patients with thymoma without myasthenia gravis: A systematic review and meta-analysis
Source: Front Oncol. 2023 Feb 8;13:1061264. doi: 10.3389/fonc.2023.1061264 (PMC9944936; doi:10.3389/fonc.2023.1061264)
Supplement: Supplementary file 1 [file Table_1.docx]

Supplementary Table 1. The surgical procedures in enrolled investigations

| **Study, year** | **Surgical procedure** |
| --- | --- |
| Li 2004 | NR |
| Kondo 2005 | Extended thymectomy：275 Thymectomy：321 Tumor resection：137 |
| Nakajima 2008 | Thymectomy:55 |
| Yamada 2015 | Extended thymectomy: 109 Thymectomy and tumor resection: 14 |
| Sun 2010 | Extended thymectomy：46 Thymectomy：58 Tumor resection：21 |
| Qian 2017 | Tumor resection：56 Extended thymectomy：70 |
| Zhao 2017 | NR |
| Mineo 2018 | Resection of the neoplastic thymus and the mediastinal fatty tissue: 104 |
| Xu 2020 | Thymomectomy：51 Thymomectomy and anterior mediastinal lymph node detection：41 |
| Zhang 2020 | Thymomectomy: 43 Thymectomy: 186 |
| Kim 2021 | Extended thymectomy：187 Partial thymectomy：4 |
| Marcuse 2021 | Thymectomy:44 |
| Nabe 2021 | Tumor resection 9 Thymectomy: 10 Extended thymectomy 6 Extended pleurectomy decortication: 1 |

NR: Non-reported

Thymomectomy: resection tumor without removal of the thymus gland

Thymectomy: complete removal of both tumor and thymus

Extended thymectomy: resection of the thymoma, thymus, and anterior mediastinal adipose tissue around the thymus.

Tumor resection: resection of the tumor and a portion of the thymus
